# High Asymptomatic Carriage With the Omicron Variant in South Africa

**DOI:** 10.1093/cid/ciac237

**Published:** 2022-03-30

**Authors:** Nigel Garrett, Asa Tapley, Jessica Andriesen, Ishen Seocharan, Leigh H Fisher, Lisa Bunts, Nicole Espy, Carole L Wallis, April Kaur Randhawa, Maurine D Miner, Nzeera Ketter, Margaret Yacovone, Ameena Goga, Yunda Huang, John Hural, Philip Kotze, Linda-Gail Bekker, Glenda E Gray, Lawrence Corey, Ubuntu Study Team, Khatija Ahmed, Sharlaa Badal-Faesen, Shaun Barnabas, William Brumskine, Kim Comline, Andreas Diacon, Thozama Dubula, Katherine Gill, Coert Grobbelaar, Craig Innes, Sheetal Kassim, Sheena Kotze, Erica Lazarus, Johannes Lombaard, Angelique Luabeya, Rebone Molobane Maboa, Scott Mahoney, Disebo Mahkaza, Moelo Malahleha, Daniel Malan, Kathryn Mngadi, Nivashnee Naicker, Vimla Naicker, Logashvari Naidoo, Maphoshane Nchabeleng, Mohammed Rassool, Elizabeth Spooner, Hugo Tempelman, Nyaradzo Mgodi, Sufia Dadabhai, Joe Makhema, Harriet Nuwagaba-Biribonwoha, Taraz Samandari, Peter James Elyanu, Roma Chilengi, Zvavahera Chirenje, Julie McElrath, Myron Cohen, James Kublin, Peter Gilbert, Melissa Peda, Erica Andersen-Nissen, Guido Ferrari, Manuel Villaran, Azwidhwi Takalani, Marianne Gildea, Michelle Nebergall, Carrie Sopher, Lori Proulx-Burns, Dhevium Govender, Lisa Sanders, Jen Hanke, Kagisho Baepanye, Bert Le Roux, Haven Wilvich, Smitha Sripathy, Daciana Margineantu, Valerie Brown, Kim Linton, Haley Howell, Bianca Noronha, Sarah Nikles, Alicia Toledano, Jeanine May, Jill El-Khorazaty, Keshani Naidoo, Azwidhwi Takalani, Kentse Khuto, Fatima Mayat, Lara Fairall, Ian Sanne

**Affiliations:** Centre for the AIDS Programme of Research in South Africa, University of KwaZulu–Natal, Durban, South Africa; Vaccine and Infectious Disease Division, Fred Hutchinson Cancer Research Center, Seattle, Washington, USA; University of Washington School of Medicine, Seattle, Washington, USA; Vaccine and Infectious Disease Division, Fred Hutchinson Cancer Research Center, Seattle, Washington, USA; South African Medical Research Council, Cape Town, South Africa; Vaccine and Infectious Disease Division, Fred Hutchinson Cancer Research Center, Seattle, Washington, USA; Vaccine and Infectious Disease Division, Fred Hutchinson Cancer Research Center, Seattle, Washington, USA; Vaccine and Infectious Disease Division, Fred Hutchinson Cancer Research Center, Seattle, Washington, USA; Bio Analytical Research Corporation South Africa and Lancet Laboratories, Johannesburg, South Africa; Vaccine and Infectious Disease Division, Fred Hutchinson Cancer Research Center, Seattle, Washington, USA; Vaccine and Infectious Disease Division, Fred Hutchinson Cancer Research Center, Seattle, Washington, USA; Vaccine and Infectious Disease Division, Fred Hutchinson Cancer Research Center, Seattle, Washington, USA; National Institute of Allergy and Infectious Diseases, National Institutes of Health, Bethesda, Maryland, USA; South African Medical Research Council, Cape Town, South Africa; University of Pretoria, Pretoria, South Africaand; Vaccine and Infectious Disease Division, Fred Hutchinson Cancer Research Center, Seattle, Washington, USA; Department of Global Health, University of Washington, Seattle, Washington, USA; Vaccine and Infectious Disease Division, Fred Hutchinson Cancer Research Center, Seattle, Washington, USA; Qhakaza Mbokodo Research Clinic, Ladysmith, South Africa; Desmond Tutu HIV Centre, University of Cape Town, Cape Town, South Africa; South African Medical Research Council, Cape Town, South Africa; Vaccine and Infectious Disease Division, Fred Hutchinson Cancer Research Center, Seattle, Washington, USA

**Keywords:** Omicron variant, asymptomatic carriage, South Africa, SARS-CoV-2, PWH

## Abstract

We report a 23% asymptomatic severe acute respiratory syndrome coronavirus 2 (SARS CoV-2) Omicron carriage rate in participants being enrolled into a clinical trial in South Africa, 15-fold higher than in trials before Omicron. We also found lower CD4^ + ^T-cell counts in persons with human immunodeficiency virus (HIV) strongly correlated with increased odds of being SARS-CoV-2 polymerase chain reaction (PCR) positive.

The emergence of the B.1.1.529 (Omicron) severe acute respiratory syndrome coronavirus 2 (SARS-CoV-2) variant, first identified in Botswana and South Africa [1] and now detected in over 165 countries, has led to a new global wave of coronavirus disease 2019 (COVID-19). In mid-November 2021, South Africa experienced a new rise in COVID-19 cases at a rate faster than any of the 3 previous waves [2], even in settings of ongoing mask mandates and high prevalence of prior infection [3, 4]. The early widespread dissemination of Omicron globally indicated a need to better understand the transmission dynamics of Omicron, including asymptomatic spread among immunocompetent and immunocompromised populations.

## METHODS

On 2 December 2021, we began enrolling participants into the Ubuntu multicenter Phase 3 clinical trial in sub-Saharan Africa (with all sites initially enrolling located in South Africa) to assess the relative risk of the COVID-19 mRNA vaccine mRNA-1273 between study groups in persons (adults) with human immunodeficiency virus (HIV, PWH) or another comorbidity known to be associated with severe COVID-19 (CoVPN 3008, NCT05168813). A smaller number of HIV negative persons was also included. Previously vaccinated individuals were excluded. Baseline testing included HIV screening, CD4^ + ^T-cell count and HIV viral load (if HIV positive), and collection of a nasal swab for SARS-CoV-2 reverse transcriptase polymerase chain reaction (RT-PCR) testing with the Abbott Real-Time SARS-CoV-2 assay (Abbott Laboratories, Abbott Park, IL, USA). The Assure Ecotest IgG/IgM Rapid Test (Assure Tech, Hangzhou, China) was used to determine baseline SARS-CoV-2 antibody status at screening. TaqPath™ COVID 19 CE IVD RT PCR (ThermoFisher, Waltham, Massachusetts, USA) was used to amplify Orf and N genes. Study participants had to be clinically well with no signs/symptoms of COVID-19 to be vaccinated upon enrollment. Results from the Ubuntu trial were compared with results of participants in the Sisonke sub-study and Ensemble 1 trials.

The Ubuntu, Sisonke, and Ensemble 1 trials were approved by the South African Health Products Regulatory Authority (SAHPRA) and by all local South African research ethics committees. Patient/participant consent was obtained, and the appropriate institutional forms have been archived.

## RESULTS

As of 20 January 2022, a total of 1172 adults were enrolled in the Ubuntu trial across eight South African provinces (see Supplementary Methods for list of clinical research sites). The median age of participants was 40 years (range 18–76 years), and 76% were assigned female sex at birth.

**Figure 1.**
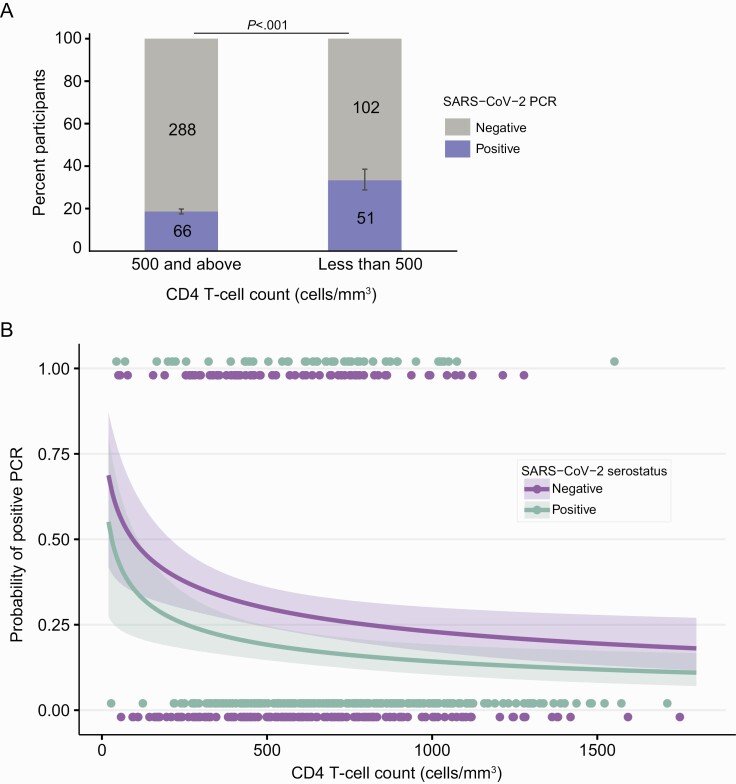
*A*, Percent of PWH positive for SARS-CoV-2 PCR (*blue*) among those with CD4^ + ^T-cell counts ≥500 and <500 cells/mm^3^. Error bars show 95% CI. Actual numbers of individuals in each group are displayed on the bar graphs. *B*, Probability of PCR positivity by CD4^ + ^T-cell count among PWH. Dots display observed data in baseline seronegative (*purple*) and seropositive (*green*) asymptomatic study participants. Positive and negative PCR results are plotted around 1 and 0 with a small vertical offset by serostatus for clarity. Lines and shaded regions display the estimated probability of positive PCR and corresponding 95% CIs, respectively. Logistic regression was used to model the probability of PCR positivity by CD4 count, adjusting for baseline serostatus. Abbreviations: CI, confidence interval; PCR, polymerase chain reaction; PWH, people with human immunodeficiency virus; SARS-CoV-2, severe acute respiratory syndrome coronavirus 2.

Baseline nasal swab data were available for 719/1172 (61.3%) enrolled participants: 162 (23%) of whom had asymptomatic SARS-CoV-2 infection by RT-PCR (Supplementary Table 1). Province-wide prevalence data can be found in Supplementary Table 2. SARS-CoV-2 infection was more frequent among SARS-CoV-2 seronegative (95/317) compared to seropositive (67/402) participants, regardless of baseline HIV status (30% vs 17%, *P* < .001) (Supplementary Table 2). SARS-CoV-2 infection was detected among 51/153 (33%) of PWH with a CD4^ + ^T-cell count <500 cells/mm^3^ vs 66/354 (19%) of PWH with counts ≥500 cells/mm^3^ (*P* < .001) (Figure 1A), an association seen in both SARS-CoV-2 seropositive and seronegative PWH (Supplementary Table 3). A 10-fold decrease in CD4 count corresponded to a 3.2-fold higher odds of a positive PCR test, after adjusting for serostatus (95% confidence interval [CI]:1.16-3.03 fold higher, *P* = .003, Figure 1B). Although all SARS-CoV-2 cases were identified during the Omicron outbreak, 62 of the initial 72 infections underwent additional testing to evaluate S gene dropout, and the Orf and N genes were successfully amplified for 56 of these samples. All 56 had S gene dropout, suggestive of Omicron infection [5]. Sequencing was successful for 38 of these 56 samples, and all were confirmed as Omicron. The median RT-PCR cycle threshold (Ct) value was 25.8 (range 14.4–34.9), with Ct values <25 in 27/56 (48%) and ≤20 in 10/56 (18%) of participants (Supplementary Figure 1; Supplementary Table 4).

Symptom data were available for 87/162 SARS-CoV-2 positive participants for the 7-day period following the positive PCR result: 52/87 (60%) remained without symptoms, of whom 18 were seropositive and 34 seronegative (Supplementary Table 5).

Nasal swab sampling at the initial vaccination visit has been used in several COVID-19 vaccine efficacy trials to define persons infected at the time of study entry [6–9]. Studies conducted before Omicron consistently exhibited asymptomatic carriage of pre-Omicron variants in <2% of participants at such visits [6, 7, 9] (Supplementary Table 1), including a 1227 PWH subgroup in the Ensemble 1 study [7], largely enrolled during the Beta outbreak in South Africa. In addition to these studies, the Sisonke study [8], conducted exclusively in South Africa between June and August 2021 during the Beta and Delta outbreaks, demonstrated an asymptomatic carriage rate of 2.4% in the subgroup sampled on day of vaccination. Of the 577 participants of the Sisonke subgroup resampled from mid-November to 7 December 2021, at the 6-month follow-up visit (time of Omicron outbreak), 91/577 (16%) participants had SARS-COV-2 detected in their nasal swab sample despite prior vaccination (Supplementary Table 6). In this cohort, the frequency of PCR positivity with Omicron was similar between PWH (27/169: 16%) and HIV negative participants (62/405: 15.3%).

## DISCUSSION

The prevalence of asymptomatic infection seen in our study (23%) strongly suggests that Omicron has a much higher rate of asymptomatic carriage than other variants of concern (VoC). Studies prior to Omicron have shown that 30-40% of SARS-CoV-2 infections are asymptomatic [10, 11]. Our data suggest that a much higher proportion of Omicron infections may be asymptomatic; data consistent with the pattern of milder disease seen with Omicron in general. Importantly, many of these asymptomatic carriers identified in our study and others had high nasal viral titers, as indicated by the relatively low RT-PCR Ct values [12]. The high prevalence of cryptic carriage of Omicron helps explain its widespread dissemination globally, presenting a substantial challenge to many current infection control strategies, including symptom-triggered testing, contact tracing, and masking policies.

Our findings that large numbers of PWH could be at elevated risk of shedding Omicron and that lower CD4^ + ^T-cell counts may be associated with an increased likelihood of being PCR positive for SARS-CoV-2 are both provocative and perhaps conceptually obvious. The data are provocative because the cutoff point was seen at the relatively high level of <500 cells/mm^3^, although more data are needed to validate this finding. Prior studies largely conducted during ancestral strain time periods have not found an increased prevalence of SARS-CoV-2 among PWH compared to HIV-uninfected people [13–16]. VoC that exhibit levels of resistance to the immunodominant epitopes associated with immune protection may be a factor in these differences [17]. Our findings do not differentiate whether PWH with CD4 counts <500 cells/mm^3^ have a greater risk of acquiring SARS-CoV-2, or whether their high subclinical prevalence of Omicron infection is related to impaired clearance of the virus. Persistent viral replication is of significant public health importance both in terms of transmission risk as well as the risk of viral evolution and generation of further VoC. Studies of viral persistence and viral variation among PWH are needed. Persistent SARS-CoV-2 viral shedding may not simply be limited to individuals off treatment and with the most advanced HIV disease but could also impact many PWH on antiretroviral therapy (ART) with more controlled disease.

Study limitations include unclear generalizability outside of South Africa, particularly to places with lower SARS-CoV-2 seroprevalence. Furthermore, the interpretation of positive SARS-CoV-2 serology tests was limited by the fact that they could not differentiate between which strains led to the antibodies (eg, Delta, Omicron) or when the infections took place—including potentially ongoing infections.

The widespread asymptomatic carriage found with Omicron indicates that investment in second-generation vaccines to prevent infection, not only disease, and enhanced surveillance with rapid testing paired with real-time whole genome sequencing should be explored. These data also highlight the urgent need for larger studies to better characterize how immunocompromise influences infection acquisition and clearance. The rapid dissemination of Omicron related to this high rate of subclinical infection has highlighted our interconnectedness, and research that helps us protect at-risk populations will also serve to protect us all.

## Supplementary Data

Supplementary materials are available at *Clinical Infectious Diseases* online. Consisting of data provided by the authors to benefit the reader, the posted materials are not copyedited and are the sole responsibility of the authors, so questions or comments should be addressed to the corresponding author.

ciac237_suppl_Supplementary_Material

## Notes

***Acknowledgments***. The authors thank the participants and study staff of the Ubuntu and Sisonke trial teams (see Supplementary Acknowledgments) for their dedication and contributions to the trial. They wish to thank Peter Meewes and Gillian Hunt for running assays.

***Study team***. Khatija Ahmed (Setshaba Research Centre); Sharlaa Badal-Faesen (Themba Lethu HIV Research Unit); Shaun Barnabas (FAM-CRU/Family Clinical Research Unit); William Brumskine (The Aurum Institute Rustenburg Clinical Research Centre); Kim Comline (Wits Reproductive HIV Institute CRS Ward 21); Andreas Diacon (TASK - Central, Eden, Brooklyn Chest Hospital, Delft Day Hospital, Dr Ivans Toms Clinic); Thozama Dubula (Nelson Mandela Academic Clinical Research Unit); Katherine Gill (Desmond Tutu HIV Foundation Masiphumelele Clinic); Coert Grobbelaar (The Aurum Institute Clinical Research Centre Pretoria); Craig Innes (The Aurum Institute Klerksdorp Clinical Research Centre); Sheetal Kassim (Desmond Tutu Health Foundation Clinical Trials Unit); Sheena Kotze (Synexus Stanza Clinical Research Centre); Erica Lazarus (Perinatal HIV Research Unit); Johannes Lombaard (Josha Research); Angelique Luabeya (South African Tuberculosis Vaccine Initiative, Brewelskloof Hospital); Rebone Molobane Maboa (Ndlovu Research Centre); Scott Mahoney (Desmond Tutu HIV Foundation - Emavundleni Research Centre); Disebo Mahkaza (Centre for the AIDS Programme of Research in South Africa Vulindlela Clinic); Moelo Malahleha (Synergy Biomed Research Institute); Daniel Malan (PHOENIX Pharma Ltd); Kathryn Mngadi (The Aurum Institute Tembisa Clinical Research Centre); Nivashnee Naicker (Centre for the AIDS Programme of Research in South Africa eThekwini Clinic); Vimla Naicker (South African Medical Research Council – Tongaat); Logashvari Naidoo (South African Medical Research Council – Chatsworth); Maphoshane Nchabeleng (Mecru Clinical Research Unit); Friedrich Petrick (Mzansi Ethical Research Centre); Mohammed Rassool (Clinical HIV Research Unit); Elizabeth Spooner (South African Medical Research Council - Botha's Hill); Hugo Tempelman (Ndlovu Research Centre); Nyaradzo Mgodi (University of Zimbabwe Clinical Trials Research Centre); Sufia Dadabhai (Blantyre Clinical Research Site); Joe Makhema (Botswana Harvard AIDS Institute); Harriet Nuwagaba-Biribonwoha (Eswatini Prevent Center Clinical Research Site); Taraz Samandari (Kisumu Clinical Research Site/KEMRI-CDC); Peter James Elyanu (Baylor-Uganda Clinical Research Site); Roma Chilengi (Matero Clinical Research Site); Zvavahera Chirenje (University of Zimbabwe Clinical Trials Research Centre); Julie McElrath (Fred Hutchinson Cancer Research Center); Myron Cohen (University of North Carolina at Chapel Hill); James Kublin (Fred Hutchinson Cancer Research Center); Peter Gilbert (Fred Hutchinson Cancer Research Center); Melissa Peda (Fred Hutchinson Cancer Research Center); Erica Andersen-Nissen (Hutchinson Centre Research Institute of South Africa); Guido Ferrari (Duke University); Manuel Villaran (Fred Hutchinson Cancer Research Center); Azwidhwi Takalani (Hutchinson Centre Research Institute of South Africa); Marianne Gildea (FHI 360); Michelle Nebergall (Fred Hutchinson Cancer Research Center); Carrie Sopher (Fred Hutchinson Cancer Research Center); Lori Proulx-Burns (Fred Hutchinson Cancer Research Center); Dhevium Govender (Hutchinson Centre Research Institute of South Africa); Lisa Sanders (Fred Hutchinson Cancer Research Center); Jen Hanke (Fred Hutchinson Cancer Research Center); Laurie Rinn (Fred Hutchinson Cancer Research Center); Jackline Odhiambo (Hutchinson Centre Research Institute of South Africa); Kagisho Baepanye (Hutchinson Centre Research Institute of South Africa); Bert Le Roux (Hutchinson Centre Research Institute of South Africa); Haven Wilvich (Fred Hutchinson Cancer Research Center); Smitha Sripathy (Fred Hutchinson Cancer Research Center); Daciana Margineantu (Fred Hutchinson Cancer Research Center); Valerie Brown (The Emmes Company, LLC); Kim Linton (The Emmes Company, LLC); Haley Howell (The Emmes Company, LLC); Bianca Noronha (The Emmes Company, LLC); Sarah Nikles (The Emmes Company, LLC); Alicia Toledano (The Emmes Company, LLC); Jeanine May (The Emmes Company, LLC); Jill El-Khorazaty (The Emmes Company, LLC); Keshani Naidoo (Desmond Tutu Health Foundation); Azwidhwi Takalani (Hutchinson Centre Research Institute of South Africa); Kentse Khuto (Hutchinson Centre Research Institute of South Africa); Fatima Mayat (Perinatal HIV Research Unit); Lara Fairall (University of Cape Town, King’s College London); Ian Sanne (Right to Care).

***Financial support*.** This work was supported by the National Institute of Allergy and Infectious Diseases (NIAID) of the National Institutes of Health (NIH) grants (grant numbers UM1 AI068614-14 [CoVPN LOC], UM1 AI068635 [CoVPN SDMC], and 3UM1AI068618-15S1 [CoVPN LC]). The Sisonke study was funded by the National Treasury of South Africa; the National Department of Health; Solidarity Response Fund NPC; The Michael & Susan Dell Foundation; The Elma Vaccines and Immunization Foundation (grant number 21-V0001); and the Bill & Melinda Gates Foundation (grant number INV-030342). The funders had no role in data collection and analysis or decision to publish.

***Potential conflicts of interest***. All authors: No reported conflicts of interest. All authors have submitted the ICMJE Form for Disclosure of Potential Conflicts of Interest. Conflicts that the editors consider relevant to the content of the manuscript have been disclosed.
