# Supplementary material for: High Asymptomatic Carriage With the Omicron Variant in South Africa
Source: Clin Infect Dis. 2022 Mar 30;75(1):e289–92. doi: 10.1093/cid/ciac237 (PMC9383623; doi:10.1093/cid/ciac237)
Supplement: ciac237_suppl_Supplementary_Material [file ciac237_suppl_supplementary_material.pdf]

Supplemental Material

Supplemental Acknowledgements

We would like to thank the many members of the Ubuntu Study Team, including Khatija Ahmed (Setshaba Research Centre), Sharlaa Badal-Faesen (Themba Lethu HIV Research Unit), Shaun Barnabas (FAM-CRU/Family Clinical Research Unit), William Brumskine (The Aurum Institute Rustenburg Clinical Research Centre), Kim Comline (Wits Reproductive HIV Institute CRS Ward 21), Andreas Diacon (TASK - Central, Eden, Brooklyn Chest Hospital, Delft Day Hospital, Dr Ivans Toms Clinic), Thozama Dubula (Nelson Mandela Academic Clinical Research Unit), Katherine Gill (Desmond Tutu HIV Foundation Masiphumelele Clinic), Coert Grobbelaar (The Aurum Institute Clinical Research Centre Pretoria), Craig Innes (The Aurum Institute Klerksdorp Clinical Research Centre), Sheetal Kassim (Desmond Tutu Health Foundation Clinical Trials Unit), Sheena Kotze (Synexus Stanza Clinical Research Centre), Erica Lazarus (Perinatal HIV Research Unit), Johannes Lombaard (Josha Research), Angelique Luabeya (SATVI, Brewelskloof Hospital), Rebone Molobane Maboa (Ndlovu Research Centre), Scott Mahoney (Desmond Tutu HIV Foundation - Emavundleni Research Centre), Disebo Mahkaza (CAPRISA Vulindlela Clinic), Moelo Malahleha (Synergy Biomed Research Institute), Daniel Malan (PHOENIX Pharma Ltd), Kathryn Mngadi (The Aurum Institute Tembisa Clinical Research Centre), Nivashnee Naicker (CAPRISA eThekwini Clinic), Vimla Naicker (SA Medical Research Council – Tongaat), Logashvari Naidoo (SA Medical Research Council – Chatsworth), Maphoshane Nchabeleng (Mecru Clinical Research Unit), Friedrich Petrick (Mzansi Ethical Research Centre), Mohammed Rassool (Clinical HIV Research Unit), Elizabeth Spooner (SA Medical Research Council - Botha's Hill), and Hugo Tempelman (Ndlovu Research Centre).

We would also like to thank the many others involved in supporting the Ubuntu trial including Nyaradzo Mgodi (University of Zimbabwe Clinical Trials Research Centre), Sufia Dadabhai (Blantyre CRS), Joe Makhema (Botswana Harvard AIDS Institute), Harriet Nuwagaba-Biribonwoha (Eswatini Prevent Center CRS), Taraz Samandari (Kisumu CRS/KEMRI-CDC), Peter James Elyanu (Baylor-Uganda CRS), Roma Chilengi (Matero CRS), Zvavahera Chirenje (University of Zimbabwe Clinical Trials Research Centre), Julie McElrath (Fred Hutch), Myron Cohen (UNC), James Kublin (Fred Hutch), Peter Gilbert (Fred Hutch), Melissa Peda (Fred Hutch), Erica Andersen-Nissen (HCRISA), Guido Ferrari (Duke), Manuel Villaran (Fred Hutch), Azwidhwi Takalani (HCRISA), Marianne Gildea (FHI 360), Michelle Nebergall (Fred Hutch), Carrie Sopher (Fred Hutch), Lori Proulx-Burns (Fred Hutch), Dhevium Govender (HCRISA), Lisa Sanders (Fred Hutch), Jen Hanke (Fred Hutch), Laurie Rinn (Fred Hutch), Jackline Odhiambo (HCRISA), Kagisho Baepanye (HCRISA), Bert Le Roux (HCRISA), Haven Wilvich (Fred Hutch), Smitha Sripathy (Fred Hutch), Daciana Margineantu (Fred Hutch), Valerie Brown (The Emmes Company, LLC), Kim Linton (Emmes), Haley Howell (Emmes), Bianca Noronha (Emmes), Sarah Nikles (Emmes), Alicia Toledano (Emmes), Jeanine May (Emmes), and Jill El-Khorazaty (Emmes).

We would also like to thank the many others involved in supporting the Sisonke trial including Keshani Naidoo (DTHF), Azwidhwi Takalani (HCRISA), Kentse Khuto (HCRISA), Fatima Mayat (PHRU), Lara Fairall (UCT, King’s College London), and Ian Sanne (Right to Care).

Supplemental Methods

Baseline data were obtained from participants from the following clinical research sites (CRS): Aurum Institute Klerksdorp CRS, CAPRISA eThekwini CRS, CAPRISA Vulindlela CRS, Clinical HIV Research Unit (CHRU)/Helen Joseph CRS, Emavundleni CRS, Groote Schuur HIV CRS, Isipingo CRS, Josha Research CRS, Masiphumelele Clinical Research Site (MASI), Ndlovu Research Centre CRS, Nelson Mandela Academic Research Unit CRS, Qhakaza Mbokodo Research Clinic CRS, Rustenburg CRS, Soweto - Kliptown CRS, Synergy Biomed Research Institute, Synexus Stanza Clinical Research Centre CRS, TASK Central, Tembisa Clinic 4, Tongaat CRS, University of Cape Town Lung Institute CRS, and Wits RHI Ward 21 CRS. The protocol was approved by all local Institutional Review Boards and participants gave informed consent.

Participant symptom data were considered available and used for analysis if daily symptom data were entered into the study database for at least five of the seven post-PCR positive days, or if data were entered for between one and four of the seven days and an overall assessment was completed and the outcome consistent with daily symptoms that were entered.

Participants' PCR positivity was reported by SARS-CoV-2 serostatus and $CD4^{+}$ T-cell category overall and jointly with 95% confidence intervals and compared using chi-squared tests. Logistic regression was used to assess the relationship between PCR positivity and CD4 count, adjusting for baseline serostatus. CD4 counts were modeled on the log10 scale. Results from the adjusted and interaction models are summarized below. All tests were two-sided, and p-values < 0.05 were considered statistically significant.

Model	Parameter	Est. log odds	Std. err	Est. odds	p-value
**Adjusted**	log10(CD4)	-1.18	0.40	0.31 (0.14, 0.67)	0.003
	Seronegative	2.32	1.08	10.15 (1.23, 83.83)	0.032
	Seropositive	1.73	1.11	5.66 (0.64, 50.12	0.119
**Interaction**	(Intercept)	1.44	1.40		0.303
	log10(CD4)	-0.85	0.52		0.099
	Seropositive	1.56	2.22		0.481
	Seropositive x log10(CD4)	-0.78	0.80		0.331

Supplemental Tables and Figures

Supplemental Table 1. Prevalence of asymptomatic SARS-CoV-2 infection upon entry in vaccine studies.

Trial	Vaccine (developer)	Location	Dates	Predominant variant	Total participants	SARS-CoV-2 PCR+ †	Prevalence
COVE (CoVPN 3001; NCT04470427)	mRNA-1273 (Moderna)	US	Aug-Oct 2020	Ancestral	30,022	182	0.6%
Teen COVE (NCT04649151)	mRNA-1273 (Moderna)	US	Dec 2020-Feb 2021	Ancestral	3,469	22	0.6%
Ensemble (CoVPN 3003; NCT04505722.)	Ad26.COV2.S (Janssen)	US, South Africa, S. America	Sept 2021-Jan 2020	USA: ancestral<br>South Africa: Beta<br>S. America: Gamma, ancestral, P.2	Overall: 41,273<br>PWH§: 1,227	Overall: 227<br>PWH§: 7	Overall: 0.55%<br>PWH§: 0.58%
Sisonke substudy (NCT04838795)	Ad26.COV2.S (Janssen)	South Africa	June-Aug 2021	Delta	1,604	39	2.4%
Ubuntu (CoVPN 3008; NCT05168813)	mRNA-1273 (Moderna)	South Africa	Dec 2021-ongoing	Omicron	719*	162*	23%

† At time of vaccination visit

§ Most enrollment of people with HIV (PWH) occurred in South Africa

* Participants with baseline data as of Jan 20, 2022

Asymptomatic was defined as clinically well and without COVID-19 symptoms.

Supplemental Table 2. SARS-CoV-2 PCR positivity by South African province and participant serostatus.

	Total participants	SARS-CoV-2 PCR+	Prevalence
**Serostatus**			
SARS-CoV-2 Seronegative	317	95	30%
SARS-CoV-2 Seropositive	402	67	17%
**Total**	**719**	**162**	**23%**
**Province**			
Gauteng	142	44	31%
KwaZulu Natal	291	58	20%
Eastern Cape	64	9	14%
Limpopo	18	6	33%
Western Cape	112	29	26%
Mpumalanga	57	12	21%
North West	7	2	29%
Free State	28	2	7%
**Total**	**719**	**162**	**23%**

Supplemental Table 3. SARS-CoV-2 PCR positivity by CD4$^+$ T-cell count in PWH.

CD4$^+$ T-cell count (cells/mm^3)	SARS-CoV-2 PCR positive* (n)	Total (n)	% PCR positive (95% confidence interval)
**Among total study population of PWH**			
500 and above	66	354	19% (15%, 23%)
less than 500	51	153	33% (26%, 41%)
**Total**	**117**	**507**	**23% (20%, 27%)**
**Among PWH by baseline SARS-CoV-2 serology**			
SARS-CoV-2 seronegative			
500 and above	33	145	23% (17%, 30%)
less than 500	37	92	40% (31%, 50%)
**Total**	**70**	**237**	**30% (24%, 36%)**
SARS-CoV-2 seropositive			
500 and above	33	209	16% (12%, 21%)
less than 500	14	61	23% (14%, 35%)
**Total**	**47**	**270**	**17% (13%, 22%)**

*Positive cases are assumed to be Omicron, based on initial sequencing of some cases and epidemiological data.

Supplemental Table 4. Summary of cycle threshold (Ct) values by Fisher Taqpath for those samples with S gene drop out (n=56).

Target	n	Mean Ct	SD Ct	Min Ct	Median Ct	Max Ct
N gene	56	25.8	5.3	14.5	25.8	35.7
ORF1ab	56	25.2	5.5	14.4	25.7	34.1
Average N and ORF1a	56	25.4	5.4	14.4	25.8	34.9

Note Ct values of 20 and 25 correspond to log10 viral loads of approximately 6.5 and 5, respectively.

Supplemental Table 5. Persistence of asymptomatic presentation by SARS-CoV-2 serostatus.

**Serostatus**	**Asymptomatic through 7 days follow up**	**Total**	**% Asymptomatic**
SARS-CoV-2 seronegative	34	57	60%
SARS-CoV-2 seropositive	18	30	60%
**Total**	**52**	**87**	**60%**

Supplemental Table 6. Prevalence of asymptomatic SARS-CoV-2 infection by PCR in Sisonke substudy from enrollment through 6 months.

	**Predominant variant(s)***	**No. of participants**	**SARS-CoV-2 PCR+**	**Prevalence**
**Enrollment**	Beta, Delta	1,604	39	2.4%
**Week 3**	Delta	1,447	51	3.5%
**Week 6**	Delta	1,260	25	2.0%
**Month 6**	Omicron	577	91	16%
HIV positive	*Omicron*	*169*	*27*	*16%*
HIV negative	*Omicron*	*405*	*62*	*15.3%*

*Source: https://covariants.org/per-country)
Month 6 participants sub divided by HIV status are in italics.

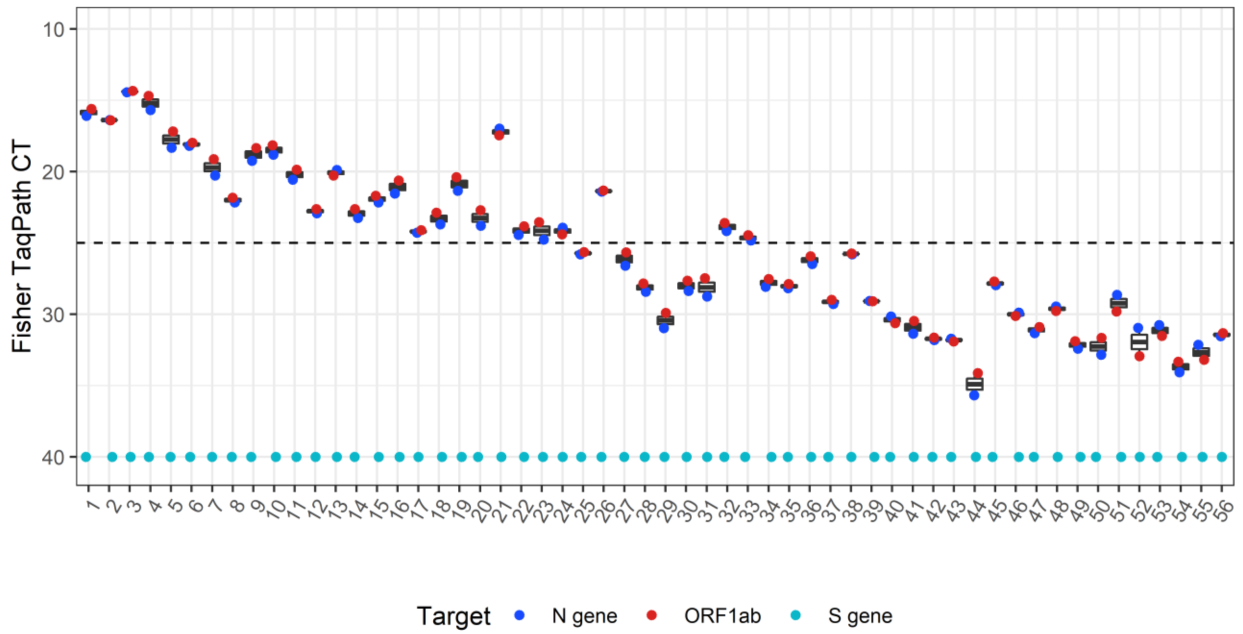

Supplemental Figure 1. Summaries of cycle threshold values. Ubuntu baseline positive swabs (x axis) with S gene target failure (n=56). Fisher Taqpath Ct arranged in order of increasing Abbott cycle number. Dotted line denotes Ct of 25. Note Ct values of 20 and 25 correspond to log10 viral loads of approximately 6.5 and 5, respectively.
